# Safety and efficacy of peptide receptor radionuclide therapy for advanced medullary thyroid cancer: a systematic review and meta-analysis

**DOI:** 10.1186/s13044-026-00290-x

**Published:** 2026-02-18

**Authors:** Ahmed Saad Abdlkadir, Dhuha Al-Adhami, Hongcheng Shi, Mike Machaba Sathekge, Areej Abu Sheikha, Issa Mohamad, Michael Kreissl, Akram Al-Ibraheem

**Affiliations:** 1 Department of Nuclear Medicine, Baghdad Radiotherapy and Nuclear Medicine Hospital, Bab Al-Muadham, Baghdad, 10047 Iraq; 2 Department of Nuclear Medicine, King Hussein Cancer Center (KHCC), Queen Rania Street, Al Jubeiha, Amman, 11941 Jordan; 3 Department of Nuclear Medicine, Zhongshan Hospital, Fudan University, Shanghai, China; 4 Department of Nuclear Medicine, University of Pretoria & Steve Biko Academic Hospital, Pretoria, 0001 South Africa; 5 Nuclear Medicine Research Infrastructure (NuMeRI), Steve Biko Academic Hospital, Pretoria, 0002 South Africa; 6 Department of Nuclear Medicine, Steve Biko Academic Hospital, Pretoria, South Africa; 7 Department of Medical Oncology, King Hussein Cancer Center (KHCC), Amman, Jordan; 8 Department of Radiation Oncology, King Hussein Cancer Center (KHCC), Amman, Jordan; 9 Division of Nuclear Medicine, Department of Radiology and Nuclear Medicine, University Hospital of Magdeburg, 39120 Magdeburg, Germany; 10 School of Medicine, University of Jordan, Amman, Jordan

**Keywords:** Medullary thyroid carcinoma, Targeted radionuclide therapy, PRRT, [^177^Lu]Lu-DOTATATE, [^90^Y]Y-DOTATOC

## Abstract

This systematic review and meta-analysis evaluated the safety and efficacy of somatostatin receptor (SSTR) peptide receptor radionuclide therapy (PRRT) in patients with metastatic or progressive medullary thyroid cancer (MTC). PubMed, Scopus, and Web of Science databases were systematically searched from inception to April 22, 2025, to identify relevant clinical studies. Methodological quality was assessed via the National Institutes of Health Quality Assessment Tool. Pooled estimates of the disease control rate (DCR), overall response rate (ORR), and adverse events (AEs) following SSTR PRRT administration were calculated for both the imaging and biochemical endpoints via Stata software version 17. The analysis revealed pooled DCRs of 52% (95% CI: 43–61%) and 58% (95% CI: 46–70%) for the biochemical and imaging endpoints, respectively. The pooled ORRs were 32% (95% CI: 23–42%) for the biochemical endpoints and 17% (95% CI: 7–26%) for the imaging endpoints. Compared with [^90^Y]Y-DOTATOC, [^177^Lu]Lu-DOTATATE had a slightly greater pooled imaging DCR (64% vs. 50%) and a lower overall toxicity rate (7% vs. 24%). The biochemical DCRs were comparable (51–52%), although [^177^Lu]Lu-DOTATATE achieved a higher biochemical ORR (37% vs. 29%). The [^90^Y]Y-DOTATOC group reported relatively higher rates of gastrointestinal and hematologic toxicities, with only limited cases of high-grade events reported in three studies. [^177^Lu]Lu-DOTATATE showed no renal toxicity, unlike [^90^Y]Y-DOTATOC, which mainly caused low-grade nephrotoxicity events at a pooled rate of 6%. Overall, this meta-analysis suggests that SSTR PRRT is a promising therapeutic option for metastatic or progressive MTC, achieving reliable disease control with a favorable safety profile.

**Supplementary Information:**

The online version contains supplementary material available at 10.1186/s13044-026-00290-x.

## Introduction

Medullary thyroid cancer (MTC) is a rare neuroendocrine neoplasm (NEN) arising from the parafollicular cells of the thyroid and represents only 1–2% of all thyroid cancers [1]. It is far less common than differentiated thyroid carcinomas and occurs in sporadic (75–80%) and familial (20–25%) forms. Sporadic MTC typically affects older adults without a family history, whereas familial MTC is often associated with multiple endocrine neoplasia type 2 [2].

Surgical resection of locally confined MTC is generally curative; however, a subset of tumors demonstrates aggressive behavior [3]. In patients with progressive/metastatic MTC, management remains challenging. Cytotoxic chemotherapy has shown limited efficacy, with objective response rates (ORRs) typically less than 20% [4, 5]. Among systemic therapies, the tyrosine kinase inhibitors (TKIs) Vandetanib and Cabozantinib have been approved by both the Food and Drug Administration (FDA) and the European Medicine Agency (EMA) for progressive/metastatic MTC [6, 7]. Nevertheless, both agents are associated with substantial toxicity, with high-grade adverse events reported in more than 36% of patients, underscoring the need for alternative systemic treatments [6, 7].

Recent advances in radiotheranostics have led to the development and clinical adoption of somatostatin receptor (SSTR)-targeted agents for the diagnosis and treatment of NENs [8, 9]. Under this concept, [^177^Lu]Lu-DOTATATE received FDA and EMA approval following the positive results of the NETTER-1 trial in gastroenteropancreatic NENs [10]. As in other progressive/metastatic NENs, SSTR peptide receptor radionuclide therapy (PRRT) may represent a promising therapeutic approach in MTC. While several narrative reviews have suggested potential benefits [11–13], no systematic review or meta-analysis has yet synthesized the available evidence on the safety and efficacy of SSTR PRRT specifically for progressive/metastatic MTC. This systematic review and meta-analysis aims to address this gap.

## Materials and methods

This review was registered in the International Prospective Register of Systematic Reviews (Prospero ID: CRD42022350984). The meta-analysis was conducted in accordance with the most recent Preferred Reporting Items for Systematic Reviews and Meta-Analyses (PRISMA) 2020 guidelines (Table S1) [14].

### Data sources and search strategy

The PubMed, Scopus, and Web of Science databases were systematically searched from inception to April 22, 2025, to identify relevant studies evaluating the safety and efficacy of SSTR PRRT in advanced MTC. Three authors (ASA, DAA, and AA-I) independently conducted the searches via MeSH- and Emtree-based keywords to ensure comprehensive coverage of the topic (Table S2) [15]. Eligible studies were restricted to original research articles specifically assessing the safety and efficacy of SSTR PRRT in patients with MTC in clinical settings. The exclusion criteria included duplicate publications, book chapters, case reports, case series, review articles, conference proceedings, meeting abstracts, preclinical studies, and unrelated articles. All retrieved references were imported into Microsoft Excel Professional Plus 2024 (Microsoft Corp., Redmond, WA, USA) for organization, sorting, screening, and filtering.

### Data collection

A comprehensive retrieval and analysis of studies meeting the predefined inclusion criteria for this systematic review and meta-analysis was performed. A dedicated Microsoft Excel spreadsheet was developed to enable a structured and detailed examination of the selected articles. From each study, relevant data were meticulously extracted, including the primary author’s name, year of publication, country of correspondence, article type, research design, patient cohort size, modality used for baseline imaging, median patient age, prior lines of therapy, name of the SSTR PRRT agent, number of SSTR PRRT cycles, SSTR PRRT treatment strategy and administered dose, biochemical markers, interim imaging evaluation results, and response criteria applied for both biochemical and imaging endpoints (Table S3).

### Quality assessment

The quality of the included studies was thoroughly assessed via the National Institutes of Health (NIH) Quality Assessment Tool for Observational Cohorts and Cross-Sectional Studies. Three authors (ASA, DAA, and AA-I) utilized this comprehensive evaluation, which was based on 14 criteria, to examine each study’s methodological strengths and limitations [16]. We adopted the NIH quality scoring system, which categorizes studies into three distinct quality levels on the basis of numerical scores. Studies classified as “good” achieved scores between 9 and 14 points, those rated as “fair” received scores between 5 and 8 points, and studies in the “poor” category scored between 0 and 4 points.

### Statistical analysis

Pooled estimates of proportions—including the disease control rate (DCR), ORR, partial response (PR) rate, stable disease (SD) rate, complete response (CR) rate, and adverse event rate—were reported as point estimates with corresponding 95% confidence intervals (CIs). The efficacy and safety of SSTR PRRT agents, both collectively and individually, were evaluated and presented in forest plots, provided that a minimum of two studies were available for the respective domain [17]. For domains with fewer than five studies, a fixed-effects model was applied; when five or more studies were available, a random-effects model was used [17].

Publication bias was assessed via Egger’s test when at least three studies were available [18]. In cases with ten or more studies, Doi plots and the Luis Furuya–Kanamori (LFK) index were used to visually evaluate publication bias [19]. For analyses conducted with a random-effects model, between-study heterogeneity was quantified via the inconsistency index (I²), with values < 50% considered low to moderate and values > 50% indicative of substantial to considerable heterogeneity [20]. When significant heterogeneity was observed, meta-regression or subgroup analyses were performed, provided that a minimum of ten studies were available for the given domain [21]. A p value < 0.05 was considered to indicate statistical significance. All the statistical analyses were performed via Stata, version 17.0 (StataCorp LLC, College Station, TX, USA).

## Results

**Fig. 1:**
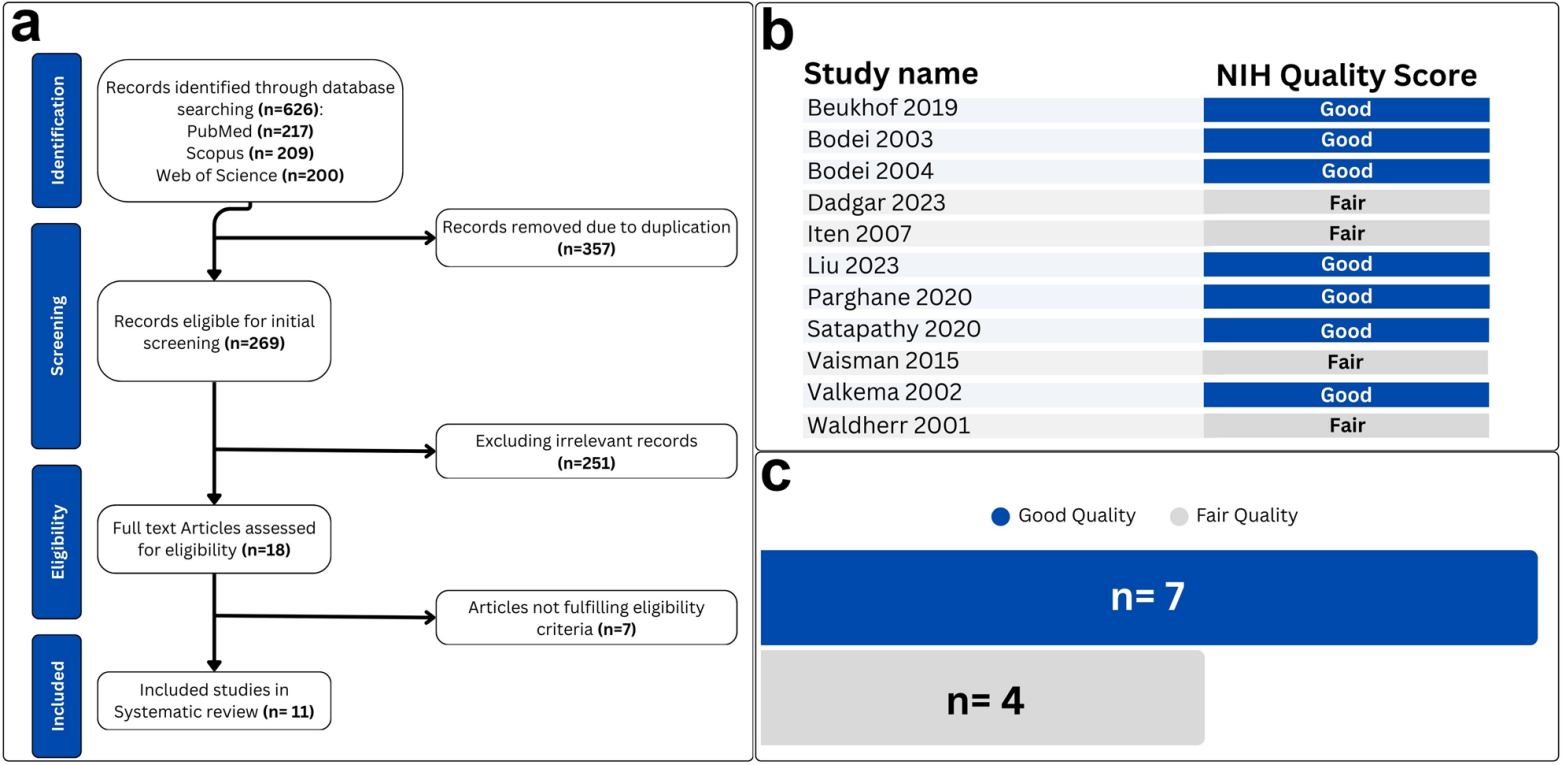
(**a**) graphical depiction of the preferred reporting items for systematic review and meta-analysis protocols (PRISMA) flowchart. (**b**, **c**) Findings pertaining to the NIH methodological quality assessment tool for observational cohort and cross-sectional studies

This systematic review initially retrieved 626 articles from three databases (PubMed: 217; Scopus: 209; Web of Science: 200). After the removal of 357 duplicates, 269 titles and abstracts were screened, with most records excluded for not meeting the study objectives. Ultimately, 11 articles fulfilled the eligibility criteria and were included in the final analysis (Fig. 1a) [22–32].

### Quality assessment

The methodological quality of the 11 studies included in this systematic review and meta-analysis was appraised via the NIH Quality Assessment Tool. Seven studies fulfilled the criteria for “good” quality [22–24, 27–29, 31], whereas the other four were deemed as “fair” (Fig. 1b, c) [25, 26, 30, 32]. The detailed results of the quality assessment are outlined in Table S4.

### Systematic review

This systematic review included 11 studies published between 2003 and 2023, comprising 498 SSTR PRRT cycles administered to 177 MTC patients [22–32]. The median age of the patients in the study cohort ranged from 35 to 62 years, with a slight male predominance (57%). Nine studies employed a retrospective design [22–25, 27–29, 31, 32], whereas two adopted a prospective design [26, 30]. Familial MTC was reported in only 15 patients across four studies [24, 28, 29, 32], whereas the majority (> 91%) had the sporadic form of the disease.

Baseline imaging utilized [^111^In]In-octreotide in seven studies [22–24, 26, 30–32], and [^68^Ga]Ga-DOTA–peptides in the remaining four [25, 27–29]. Krenning score was reported and applied to assess baseline molecular uptake in nine studies [22, 24–29, 31, 32]. Most studies (*n* = 8, 73%) administered SSTR PRRT in a cyclic dosage format, whereas a smaller proportion (*n* = 3, 27%) used a fractionated dosing regimen [26, 31, 32]. Imaging responses were evaluated via conventional imaging in six studies [22–24, 30–32], and via molecular imaging (PET or SPECT) in five studies [25–29]. The biochemical response assessment was based on changes in the serum thyrocalcitonin (TC) and carcinoembryonic antigen (CEA) levels [22–32]. Safety was reported according to the Common Terminology Criteria for Adverse Events (CTCAE) in nine studies [22, 25–32], and the World Health Organization (WHO) toxicity criteria in the remaining two [23, 24]. Geographically, seven studies were conducted in Europe [22–24, 26, 27, 31, 32], two in India [28, 29], and one each in Iran and Brazil [25, 30]. Table 1 summarizes the main characteristics of the included studies.

**Table 1:** Essential criteria for articles eligible for inclusion in a systematic review

Study Name	Country	Study Type	Sample Size	Age	Baseline Imaging	Imaging Response Criteria	Toxicity Criteria	Biochemical Response Criteria
Beukhof 2019	NL	OR	10 (M4, F6)	62	SPECT	RECIST	CTCAE 4.0	TC
Bodei 2003	IT	OR	7 (M6, F1)	47	SPECT	WHO	WHO	TC
Bodei 2004	IT	OR	21 (M13, F8)	53	SPECT	SWOG	WHO	TC
Dadgar 2023	IR	OR	6 (M3, F3)	50.6	SPECT(*n* = 2), PET(*n* = 4)	RECIST	CTCAE 4.0	TC
Iten 2007	CH	OP	31 (M10, F21)	56.7	SPECT	SC	CTCAE 3.0	SC
Liu 2023	DE	OR	25 (M14, F11)	49	PET	RECIST	CTCAE 5.0	TC
Parghane 2019	IN	OR	43 (M35, F8)	48	PET	RECIST	CTCAE 5.0	TC
Satapathy 2020	IN	OR	8 (M3, F5)	47.5	PET	RECIST	CTCAE 5.0	TC
Vaisman 2015	BR	OP	9 (M3, F6)	35.5	SPECT	RECIST	CTCAE 3.0	TC
Valkema 2002	NL	OR	5 (M3, F2)	54.6	SPECT	SWOG	CTCAE 2.0	TC
Waldherr 2001	CH	OR	12 (M7, F5)	60	SPECT	RECIST	CTCAE 2.0	TC

Abbreviations: BR, Brazil; CH, Switzerland; CTCAE, Common Terminology Criteria for Adverse Events; DE, Germany; EORTC, European Organization for Research and Treatment of Cancer; F, female; IN, India; IR, Iran; IT, Italy; KS, Karnofsky score; M, male; NL, Netherlands; NR, not reported; OP, Original prospective; OR, Original retrospective; PET, positron emission tomography; PRRT, peptide receptor radionuclide therapy; RECIST, Response Evaluation Criteria in Solid Tumors; SC, specific criteria (Detailed in Table S3); SPECT, single photon emission computed tomography; SWOG, Southwestern Oncology Group criteria; TC, Traditional criteria; WHO, World Health Organization

## Meta-analysis

### Biochemical efficacy of SSTR PRRT

**Fig. 2:**
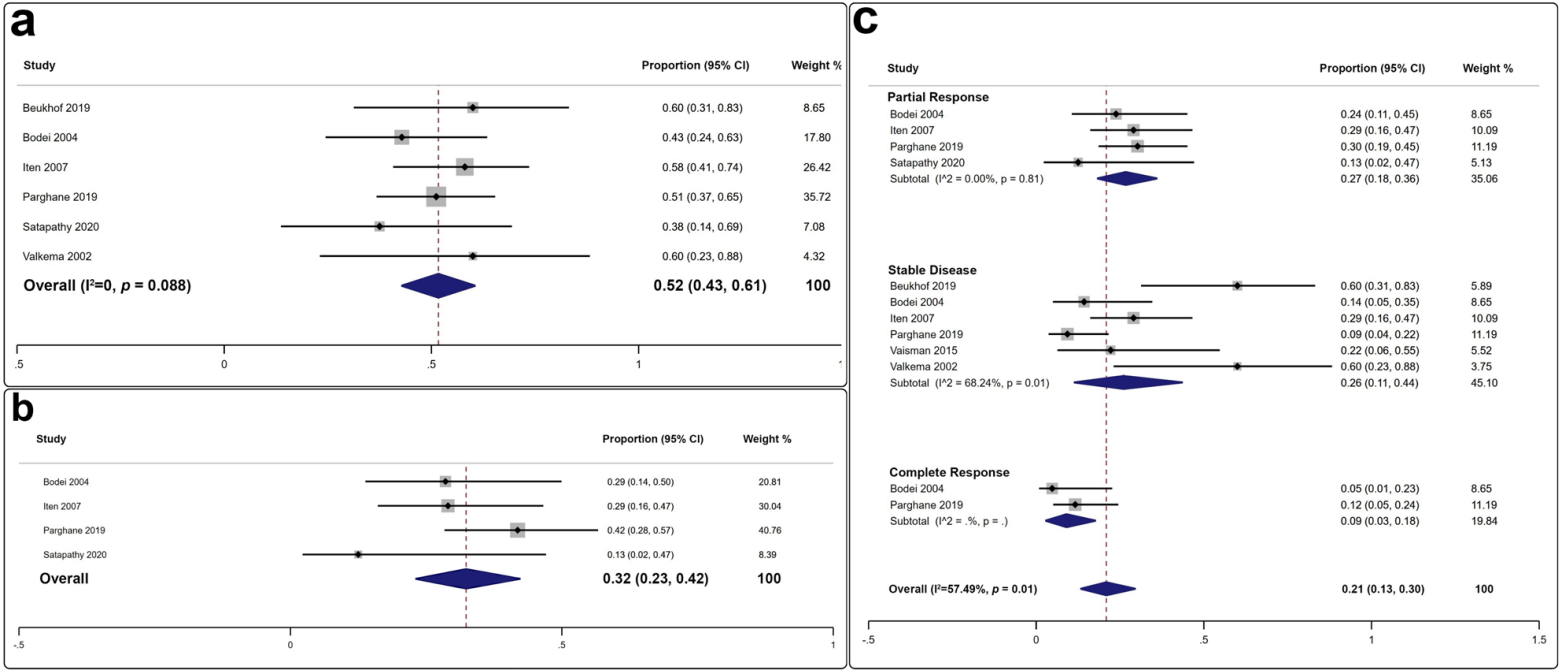
Forest plots illustrating the overall biochemical efficacy of PRRT in MTC: (**a**) Disease control rate; (**b**) Overall response rate; (**c**) Rates of stable disease, partial response, and complete response

A total of six studies reported effective biochemical disease control following the administration of 362 SSTR PRRT cycles to 118 MTC patients (Fig. 2a) [22, 23, 26, 28, 29]. The pooled DCR was 52% (95% CI: 43–61%), with no significant heterogeneity or evidence of publication bias (*p* > 0.76 for both). Four studies reported effective biochemical responses after 302 SSTR PRRT cycles were administered to 103 patients (Fig. 2b), yielding a pooled ORR of 32% (95% CI: 23–42%) [23, 26, 28, 29]. No significant publication bias was observed (*p* = 0.81). Overall, PR emerged as the most frequent posttherapy biochemical outcome, achieved in 27% (95% CI: 18–36%) of patients, followed by SD and CR (Fig. 2c).

### Imaging efficacy of SSTR PRRT

**Fig. 3:**
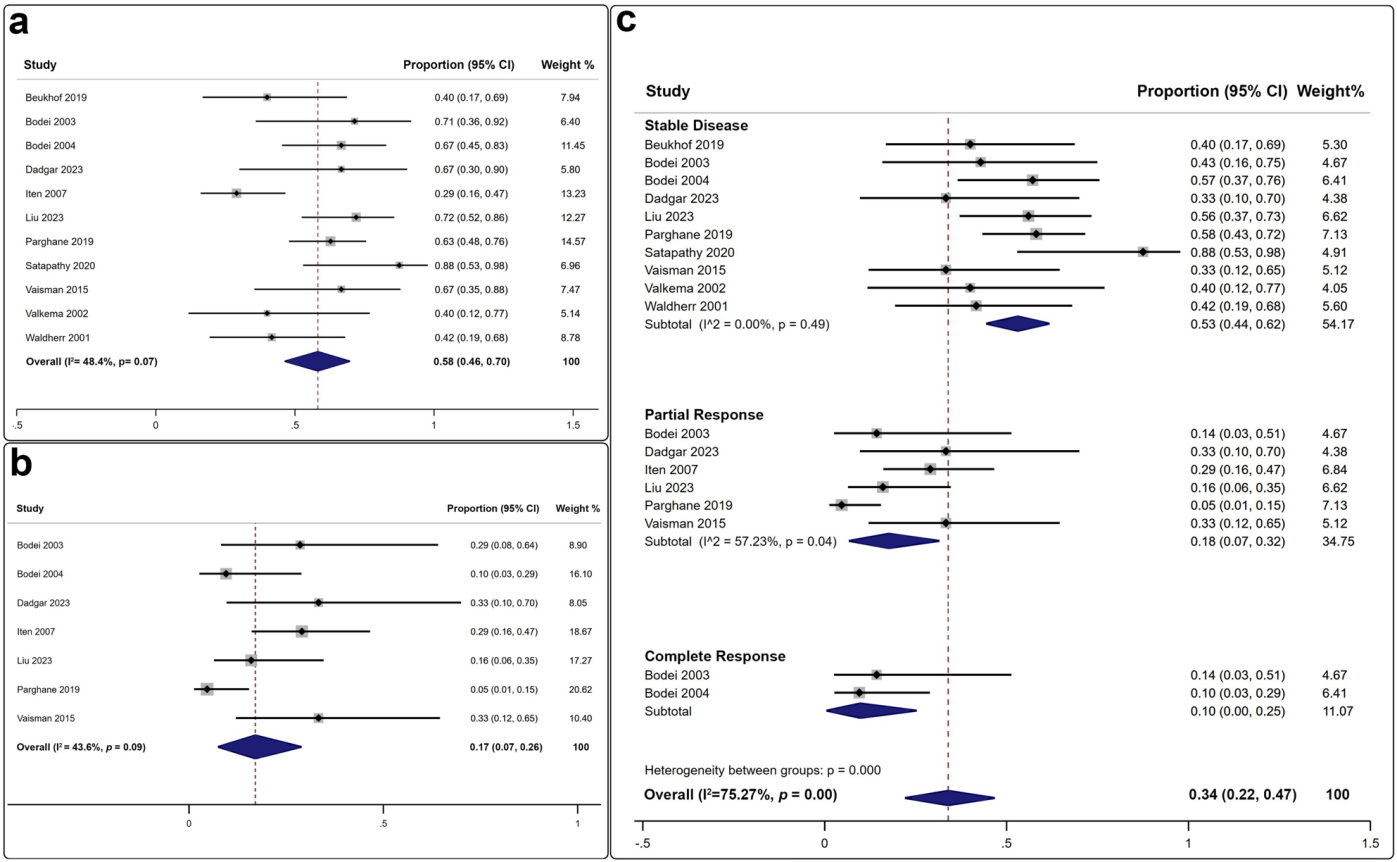
Forest plots illustrating the overall imaging efficacy of PRRT in MTC: (a) Disease control rate; (b) Overall response rate; (c) Rates of stable disease, partial response, and complete response

A total of 11 studies reported effective disease control, as assessed by either conventional or molecular imaging modalities, following the administration of 498 SSTR PRRT cycles to 177 MTC patients (Fig. 3a) [22–32]. The pooled DCR was 58% (95% CI: 46–70%), with no significant heterogeneity (I² = 48.4%) or evidence of publication bias (*p* > 0.07 for both). Seven studies reported effective imaging responses after 392 SSTR PRRT cycles were administered to 142 patients (Fig. 3b), yielding a pooled ORR of 17% (95% CI: 7–26%). No significant publication bias was detected (I² = 43.6%, *p* = 0.09) [24–28, 30]. Notably, SD was the most frequent posttherapeutic imaging outcome, observed in a cumulative 53% (95% CI: 44–62%) of patients, followed by PR and CR (Fig. 3c).

### Safety of SSTR PRRT

**Fig. 4:**
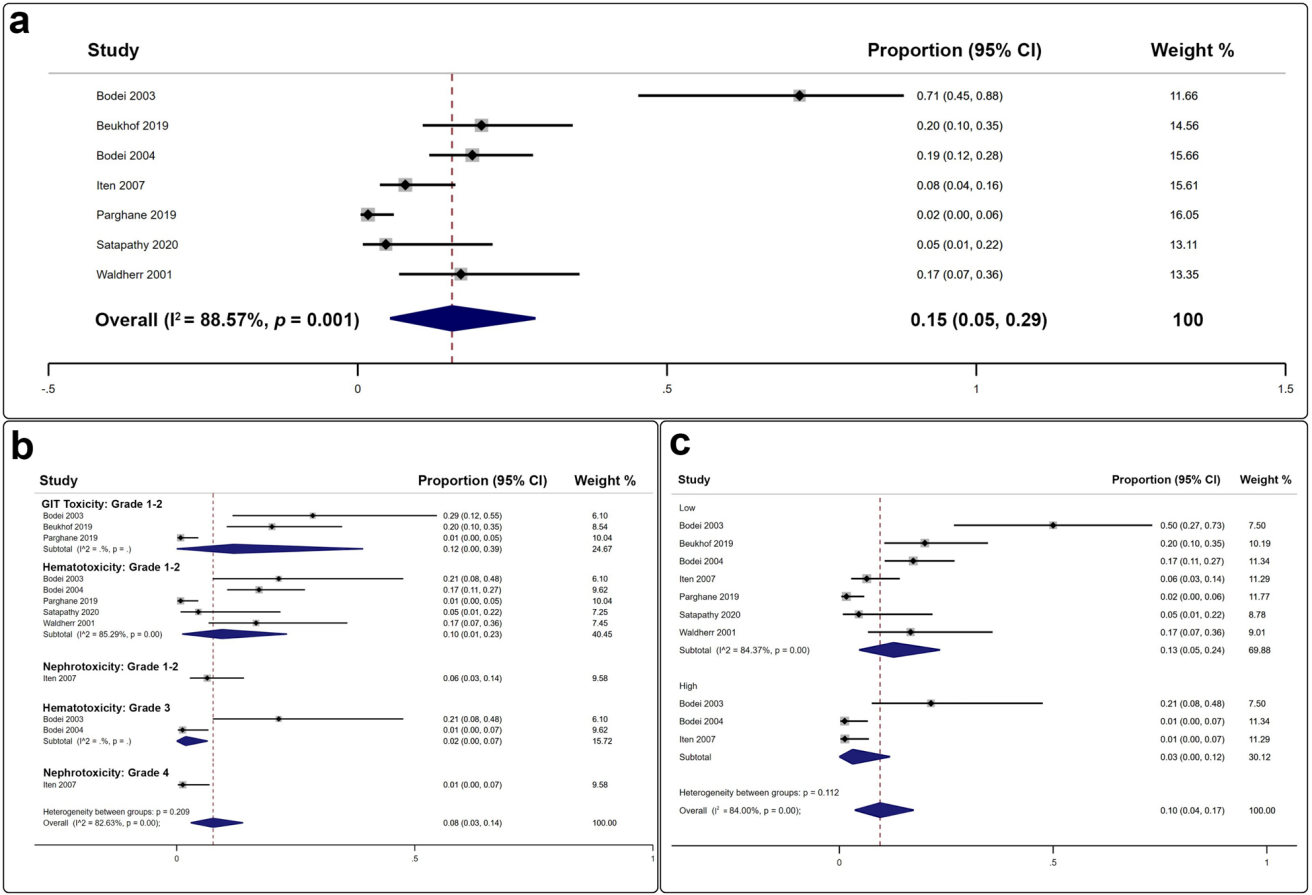
Forest plots summarizing adverse event rates following PRRT administration in MTC patients: (**a**) Pooled estimates of adverse events; (**b**) Subanalysis by adverse event category; (**c**) Subanalysis by toxicity grade

Among the 11 studies included, seven documented SSTR PRRT-associated toxicities (Fig. 4a), yielding a pooled incidence rate of 15% (95% CI: 5–29%) [22–24, 26, 28, 29, 32]. The majority of adverse effects affected the gastrointestinal and hematopoietic systems (Fig. 4b). Low-grade toxicities predominated over high-grade events (13% versus 3%). Importantly, only four instances of grade 3 hematotoxicity and one instance of grade 4 nephrotoxicity were reported (Fig. 4c).

## Subgroup meta-analysis

### Biochemical efficacy of [^177^Lu]Lu-DOTATATE

The biochemical DCR was reported in three studies, yielding a pooled estimate of 51% (95% CI: 38–64%), with no evidence of publication bias [22, 28, 29]. The pooled biochemical ORR from two studies was 37% (95% CI: 23–58%) [28, 29]. PR was achieved in 27% of the patients, SD in 26%, and CR in 12% of the patients (Table 2).

**Table 2:** Subgroup meta-analysis results of the safety and efficacy of [^177^Lu]Lu-DOTATATE

[^177^Lu]Lu-DOTATATE						
Category	Domain	Studies	Estimate (95% CI)	Heterogeneity		Egger’s
				I^2^	p-value	p-value
Imaging Response	DCR	5	0.64 (0.52–0.75)	1.4%	0.4	0.8822
	ORR	3	0.18 (0.1–0.49)	N/A	N/A	0.12
	SD	5	0.53 (0.35–0.70)	43.1%	0.13	0.58
	PR	3	0.18 (0.1–0.49)	N/A	N/A	0.12
Biochemical Response	DCR	3	0.51 (0.38–0.64)	N/A	N/A	0.86
	ORR	2	0.37 (0.23–0.58)	N/A	N/A	N/A
	SD	3	0.26 (0.02–0.61)	N/A	N/A	0.43
	PR	2	0.27 (0.15–0.40)	N/A	N/A	N/A
	CR	1	0.12 (0.05–0.24)	N/A	N/A	N/A
Toxicity	Overall	3	0.07 (0.0-0.22)	N/A	N/A	0.67
	Low-grade	3	0.07 (0.0-0.22)	N/A	N/A	0.67
	HT G1-2	2	0.01 (0.0-0.03)	N/A	N/A	N/A
	GIT G1-2	2	0.03 (0.01–0.07)	N/A	N/A	N/A

Abbreviations: CI, confidence interval; CR, complete response; DCR, disease control rate; G1-2, grade 1–2; G3, grade 3; G4, grade 4; GIT, gastrointestinal toxicity; HT, hematologic toxicity; I², inconsistency index; N/A, not available; ORR, overall response rate; PR, partial response; RT, renal toxicity; SD, stable disease

### Imaging efficacy of [^177^Lu]Lu-DOTATATE

Five studies involving [^177^Lu]Lu-DOTATATE reported disease control in MTC patients, with a pooled imaging DCR of 64% (95% CI: 52–75%), with no significant heterogeneity or evidence of publication bias (*p* > 0.4 for both) [22, 25, 28–30]. The corresponding ORR from three studies was 18% (95% CI: 10–49%) [25, 28, 30]. SD was the most common imaging outcome (53%, 95% CI: 35–70%) across the five studies, whereas PR occurred in 18% of the cases, and no complete responses were recorded in the imaging assessments (Table 2).

### Safety of [^177^Lu]Lu-DOTATATE

The pooled incidence of any-grade toxicity was 7% (95% CI: 0–22%) across the three studies [22, 28, 29]. Grade 1–2 gastrointestinal manifestations predominated at 3% (95% CI: 1–7%), followed only by Grade 1–2 hematotoxicity, which occurred in 1% (95% CI: 0–3%) of the patients. Notably, no high-grade hematologic or gastrointestinal adverse events were reported (Table 2).

### Biochemical efficacy of [^90^Y]Y-DOTATOC

Two studies assessed biochemical DCR, with a pooled value of 52% (95% CI: 43–58%) [24, 26]. The biochemical ORR was 29% (95% CI: 17–42%) [24, 26]. PR occurred in 27% of patients, SD in 23%, and CR in 5% of patients (Table 3).

**Table 3:** Subgroup meta-analysis results of the safety and efficacy of [^90^Y]Y-DOTATOC

[^90^Y]Y-DOTATOC						
Category	Domain	Studies	Estimate (95% CI)	Heterogeneity		Egger’s
				I^2^	p-value	p-value
Imaging Response	DCR	4	0.50 (0.28–0.72)	N/A	N/A	0.4294
	ORR	3	0.20 (0.08–0.37)	N/A	N/A	0.98
	SD	3	0.50 (0.34–0.66)	N/A	N/A	0.49
	PR	2	0.25 (0.12–0.42)	N/A	N/A	N/A
	CR	2	0.10 (0.1–0.25)	N/A	N/A	N/A
Biochemical Response	DCR	2	0.52 (0.43–0.58)	N/A	N/A	N/A
	ORR	2	0.29 (0.17–0.42)	N/A	N/A	N/A
	SD	2	0.23 (0.12–0.35)	N/A	N/A	N/A
	PR	2	0.27 (0.15–0.40)	N/A	N/A	N/A
	CR	1	0.05 (0.01–0.23)	N/A	N/A	N/A
Toxicity	Overall	4	0.24 (0.07–0.45)	N/A	N/A	0.23
	Low-grade	4	0.18 (0.06–0.33)	N/A	N/A	0.06
	High-grade	3	0.03 (0-0.12)	N/A	N/A	0.09
	HT G1-2	3	0.17 (0.10–0.25)	N/A	N/A	0.08
	HT G3	2	0.02 (0-0.07)	N/A	N/A	N/A
	GIT G1-2	1	0.29 (0.12–0.55)	N/A	N/A	N/A
	RT G1	1	0.06 (0.03–0.14)	N/A	N/A	N/A
	RT G4	1	0.01 (0-0.07)	N/A	N/A	N/A

Abbreviations: CI, confidence interval; CR, complete response; DCR, disease control rate; G1-2, grade 1–2; G3, grade 3; G4, grade 4; GIT, gastrointestinal toxicity; HT, hematologic toxicity; I², Inconsistency index; N/A, not available; ORR, overall response rate; PR, partial response; RT, renal toxicity; SD, stable disease

### Imaging efficacy of [^90^Y]Y-DOTATOC

Four studies of [^90^Y]Y-DOTATOC yielded a pooled imaging DCR of 50% (95% CI: 28–72%) [23, 24, 26, 32]. The ORR from three studies was 20% (95% CI: 8–37%) [23, 24, 26]. SD was observed in 50% of patients, PR in 25%, and CR in 10% (Table 3).

### Safety of [^90^Y]Y-DOTATOC

Any-grade adverse events from [^90^Y]Y-DOTATOC occurred in 24% (95% CI: 7–45%) of patients, with low-grade toxicity in 18% (95% CI: 6–33%) of patients [23, 24, 26, 32]. High-grade adverse events were infrequent at 3% (95% CI: 0–12%). The prevalence of Grade 1–2 gastrointestinal manifestations was 29% (95% CI: 12–55%). Grade 1–2 hematotoxicity was recorded in 17% of the patients, and grade 1 nephrotoxicity was recorded in 6%. High-grade toxicity was uncommon, with grade 3 hematotoxicity in 2% and grade 4 nephrotoxicity in 1% of patients (Table 3).

## Discussion

This systematic review and meta-analysis is the first to comprehensively evaluate the safety and efficacy of SSTR PRRT in metastatic or progressive MTC patients. Across 177 patients who received a total of 498 SSTR PRRT cycles, the cumulative DCR exceeded 50% according to both radiological and biochemical assessments [22–32]. Approximately one-third of patients achieved a favorable biochemical response, while the pooled radiological ORR was 17% on posttherapeutic imaging. Safety analysis indicated that low-grade gastrointestinal and hematologic toxicities predominated, with only rare instances of high-grade nephrotoxicity and hematotoxicity, confirming the favorable tolerability profile of SSTR PRRT [22–24, 26, 28, 29, 32].

Subgroup analysis of the two most frequently employed agents revealed that [^177^Lu]Lu-DOTATATE achieved a slightly higher pooled imaging DCR (64% vs. 50%) and lower overall toxicity (7% vs. 24%) than did [^90^Y]Y-DOTATOC. The biochemical DCRs were comparable (≈ 51–52%), although [^177^Lu]Lu-DOTATATE demonstrated a higher biochemical ORR (37% vs. 29%) [22–30, 32].

No nephrotoxicity events were observed following [^177^Lu]Lu-DOTATATE administration, unlike [^90^Y]Y-DOTATOC, which demonstrated a 6% pooled nephrotoxicity rate, largely within the low-grade range. The [^90^Y]Y-DOTATOC cohort experienced higher rates of gastrointestinal and hematologic toxicities, as shown in our meta-analysis. This might be attributable to its higher β-emission energy, greater tissue penetration, and increased off-target radiation—consistent with prior clinical reports [33]. However, variations in dosing regimens across studies (e.g., single vs. multiple cycles, cumulative administered activity, and use of renal-protective agents) may also influence the observed toxicity spectrum. Therefore, correlation analyses between toxicity incidence and cumulative administered activity of [^90^Y]Y-DOTATOC are warranted but currently unavailable for meta-analysis. Future investigations should aim to perform such correlation analyses to better elucidate dose–toxicity relationships and optimize therapeutic safety. Notably, we could not explore the safety and efficacy of [^111^In]in-octreotide, as the current evidence is limited to singular investigation [31].

In advanced or metastatic MTC, quality-of-life outcomes are critical complements to biochemical and radiological endpoints. Patients often experience visceral or skeletal pain, which substantially affects their functional status and well-being [34]. However, only one included study systematically assessed the impact of SSTR PRRT on QoL, underscoring a substantial evidence gap [30]. Future clinical trials should incorporate standardized QoL measures to better capture the holistic benefit of therapy.

SSTR PRRT has been established primarily in gastroenteropancreatic NENs expressing SSTR, with regulatory approval following the NETTER-1 trial [10]. For MTC, the current European Society for Medical Oncology and American Thyroid Association guidelines acknowledge limited evidence and recommend SSTR PRRT only within clinical trial settings or in patients for whom TKIs are contraindicated [35, 36]. High-quality randomized controlled trials with survival endpoints are needed to define the place of SSTR PRRT in the therapeutic algorithm.

While most of the included studies followed the conventional use of SSTR PRRT in advanced NENs, one study uniquely coadministered [^177^Lu]Lu-DOTATATE with capecitabine as a radiosensitizer, achieving the highest reported DCR (86%) [29]. This unique approach merits further prospective evaluation.

The imaging response is typically assessed via a single modality—either conventional or molecular techniques. This approach risks underestimating tumor heterogeneity, as illustrated by the study of Parghane et al., which utilized dual PET/CT with [^18^F]FDG and [^68^Ga]Ga-DOTATATE, acknowledging that reduced [^68^Ga]Ga-DOTATATE uptake does not exclude progression in other tumor subclones [28].

Limitations of the current evidence base include the absence of strict inclusion criteria in many studies. In particular, baseline imaging to confirm adequate tracer uptake—such as a Krenning score ≥ 3—was not consistently applied [22, 26, 31, 32], potentially reducing the accuracy of SSTR PRRT localization and the therapeutic effect. Furthermore, some studies included heavily pretreated patients (≥ 4 prior lines of therapy) [24, 26, 27, 32], introducing heterogeneity in tumor biology, resistance patterns, and toxicity risk. Additional limitations of our review include the predominance of retrospective, single-center studies from Europe, as well as heterogeneity in the SSTR PRRT agents evaluated (Table S5). While subgroup analyses addressed some variability, meta-regression was precluded by limited sample sizes. Collectively, these findings underscore the need for standardized eligibility criteria, harmonized imaging protocols, and adequately powered multicenter trials to fully define the role of SSTR PRRT in MTC management.

## Conclusion

STTR PRRT appears to be a promising therapeutic option for MTC patients, demonstrating consistent efficacy and a favorable safety profile. Both [^177^Lu]Lu-DOTATATE and [^90^Y]Y-DOTATOC achieved disease control in more than half of the treated patients, with [^177^Lu]Lu-DOTATATE associated with a lower incidence of adverse events and no reported high-grade toxicities, which were sporadically observed only with [^90^Y]Y-DOTATOC. Limitations of the current evidence include the predominance of retrospective, single-center studies and the absence of strict eligibility criteria, factors that may have contributed to heterogeneity across some domains and subdomains. Future research should aim to address these limitations through large-scale, prospective trials to validate these encouraging preliminary findings. 

## Supplementary Information

Below is the link to the electronic supplementary material.

Supplementary Material 1

## Data Availability

The current study data are available from the corresponding author on reasonable request.
