# Supplementary material for: Safety and efficacy of peptide receptor radionuclide therapy for advanced medullary thyroid cancer: a systematic review and meta-analysis
Source: Thyroid Res. 2026 Feb 18;19:7. doi: 10.1186/s13044-026-00290-x (PMC12918018; doi:10.1186/s13044-026-00290-x)
Supplement: Supplementary file 1 — Supplementary Material 1 [file 13044_2026_290_MOESM1_ESM.docx]

**Table S1:** Filled PRISMA Protol checkelist

| **Section and Topic** | **Item #** | **Checklist item** | **Page #** |
| --- | --- | --- | --- |
| **TITLE** | | | 1 |
| Title | 1 | Identify the report as a systematic review. | ✓ |
| **ABSTRACT** | | | 1 |
| Abstract | 2 | See the PRISMA 2020 for Abstracts checklist. | ✓ |
| **INTRODUCTION** | | | 2-3 |
| Rationale | 3 | Describe the rationale for the review in the context of existing knowledge. | ✓ |
| Objectives | 4 | Provide an explicit statement of the objective(s) or question(s) the review addresses. | ✓ |
| **METHODS** | | | 3-5 |
| Eligibility criteria | 5 | Specify the inclusion and exclusion criteria for the review and how studies were grouped for the syntheses. | ✓ |
| Information sources | 6 | Specify all databases, registers, websites, organisations, reference lists and other sources searched or consulted to identify studies. Specify the date when each source was last searched or consulted. | ✓ |
| Search strategy | 7 | Present the full search strategies for all databases, registers and websites, including any filters and limits used. | ✓ |
| Selection process | 8 | Specify the methods used to decide whether a study met the inclusion criteria of the review, including how many reviewers screened each record and each report retrieved, whether they worked independently, and if applicable, details of automation tools used in the process. | ✓ |
| Data collection process | 9 | Specify the methods used to collect data from reports, including how many reviewers collected data from each report, whether they worked independently, any processes for obtaining or confirming data from study investigators, and if applicable, details of automation tools used in the process. | ✓ |
| Data items | 10a | List and define all outcomes for which data were sought. Specify whether all results that were compatible with each outcome domain in each study were sought (e.g. for all measures, time points, analyses), and if not, the methods used to decide which results to collect. | ✓ |
|  | 10b | List and define all other variables for which data were sought (e.g. participant and intervention characteristics, funding sources). Describe any assumptions made about any missing or unclear information. | ✓ |
| Study risk of bias assessment | 11 | Specify the methods used to assess risk of bias in the included studies, including details of the tool(s) used, how many reviewers assessed each study and whether they worked independently, and if applicable, details of automation tools used in the process. | ✓ |
| Effect measures | 12 | Specify for each outcome the effect measure(s) (e.g. risk ratio, mean difference) used in the synthesis or presentation of results. | ✓ |
| Synthesis methods | 13a | Describe the processes used to decide which studies were eligible for each synthesis (e.g. tabulating the study intervention characteristics and comparing against the planned groups for each synthesis (item #5)). | ✓ |
|  | 13b | Describe any methods required to prepare the data for presentation or synthesis, such as handling of missing summary statistics, or data conversions. | ✓ |
|  | 13c | Describe any methods used to tabulate or visually display results of individual studies and syntheses. | ✓ |
|  | 13d | Describe any methods used to synthesize results and provide a rationale for the choice(s). If meta-analysis was performed, describe the model(s), method(s) to identify the presence and extent of statistical heterogeneity, and software package(s) used. | ✓ |
|  | 13e | Describe any methods used to explore possible causes of heterogeneity among study results (e.g. subgroup analysis, meta-regression). | ✓ |
|  | 13f | Describe any sensitivity analyses conducted to assess robustness of the synthesized results. | ✓ |
| Reporting bias assessment | 14 | Describe any methods used to assess risk of bias due to missing results in a synthesis (arising from reporting biases). | ✓ |
| Certainty assessment | 15 | Describe any methods used to assess certainty (or confidence) in the body of evidence for an outcome. | ✓ |
| **RESULTS** | | | 5-9 |
| Study selection | 16a | Describe the results of the search and selection process, from the number of records identified in the search to the number of studies included in the review, ideally using a flow diagram. | ✓ |
|  | 16b | Cite studies that might appear to meet the inclusion criteria, but which were excluded, and explain why they were excluded. | ✓ |
| Study characteristics | 17 | Cite each included study and present its characteristics. | ✓ |
| Risk of bias in studies | 18 | Present assessments of risk of bias for each included study. | ✓ |
| Results of individual studies | 19 | For all outcomes, present, for each study: (a) summary statistics for each group (where appropriate) and (b) an effect estimate and its precision (e.g. confidence/credible interval), ideally using structured tables or plots. | ✓ |
| Results of syntheses | 20a | For each synthesis, briefly summarise the characteristics and risk of bias among contributing studies. | ✓ |
|  | 20b | Present results of all statistical syntheses conducted. If meta-analysis was done, present for each the summary estimate and its precision (e.g. confidence/credible interval) and measures of statistical heterogeneity. If comparing groups, describe the direction of the effect. | ✓ |
|  | 20c | Present results of all investigations of possible causes of heterogeneity among study results. | ✓ |
|  | 20d | Present results of all sensitivity analyses conducted to assess the robustness of the synthesized results. | ✓ |
| Reporting biases | 21 | Present assessments of risk of bias due to missing results (arising from reporting biases) for each synthesis assessed. | ✓ |
| Certainty of evidence | 22 | Present assessments of certainty (or confidence) in the body of evidence for each outcome assessed. | ✓ |
| **DISCUSSION** | | | 9-11 |
| Discussion | 23a | Provide a general interpretation of the results in the context of other evidence. | ✓ |
|  | 23b | Discuss any limitations of the evidence included in the review. | ✓ |
|  | 23c | Discuss any limitations of the review processes used. | ✓ |
|  | 23d | Discuss implications of the results for practice, policy, and future research. | ✓ |
| **OTHER INFORMATION** | | | 1, 5 |
| Registration and protocol | 24a | Provide registration information for the review, including register name and registration number, or state that the review was not registered. | ✓ |
|  | 24b | Indicate where the review protocol can be accessed, or state that a protocol was not prepared. | ✓ |
|  | 24c | Describe and explain any amendments to information provided at registration or in the protocol. | ✓ |
| Support | 25 | Describe sources of financial or non-financial support for the review, and the role of the funders or sponsors in the review. | ✓ |
| Competing interests | 26 | Declare any competing interests of review authors. | ✓ |
| Availability of data, code and other materials | 27 | Report which of the following are publicly available and where they can be found: template data collection forms; data extracted from included studies; data used for all analyses; analytic code; any other materials used in the review. | ✓ |

**Table S2:** List of employed MeSH and Emtree terms

| Search Concept Group | Keywords You Provided | MeSH (PubMed/Medline) | Emtree (Embase) |
| --- | --- | --- | --- |
| Medullary Thyroid Cancer/Neoplasm | medullary thyroid cancer/medullary thyroid carcinoma /medullary thyroid neoplasm/medullary thyroid tumor/metastatic medullary thyroid cancer | Carcinoma, Medullary [MeSH] (Neuroendocrine C-cell tumor of thyroid) Thyroid Neoplasms [MeSH] (parent term) Neoplasm Metastasis [MeSH] (for metastatic disease) | medullary thyroid carcinoma thyroid carcinoma thyroid tumor thyroid cancer metastasis |
| PRRT | Peptide receptor radionuclide therapy (PRRT) | Receptors, Peptide [MeSH] Radionuclide Therapy [MeSH] | peptide receptor radionuclide therapy radionuclide therapy receptor targeted therapy |
| Lutetium-based | 177Lu-DOTATATE/ lutetium Lu 177 dotatate/ lutetium oxodotreotide/ DOTATATE-177Lu | Lutetium[MeSH] DOTA-Tyr3-Octreotate [Supplementary Concept Record] (synonym: DOTATATE/oxodotreotide) | lutetium oxodotreotide lutetium lu 177 dotatate 177Lu-DOTATATE |
| Yttrium-based | 90Y-DOTATOC/ 90Y-DOTA-TOC/90Yttrium-DOTA-TOC/[90Yttrium-DOTA]-TOC | Yttrium Radioisotopes [MeSH] DOTA-Tyr3-Octreotide [Supplementary Concept Record] (synonym: DOTATOC) | yttrium-90 edotreotide (90Y-DOTATOC) yttrium radioisotope 90Y-DOTA-TOC |

**Table S3:** Overview of employed criteria for response assessment

| Response Category | Imaging | | | | Traditional Biochemical Criteria | | Special Criteria |
| --- | --- | --- | --- | --- | --- | --- | --- |
|  | **RECIST 1.1 (Radiological)** | **WHO Criteria (Radiological)** | **SWOG Criteria (Radiological)** | **Specific Criteria by Iten et al.** | **Calcitonin Trend** | **CEA Trend** | **Specific Criteria by Iten et al.** |
| CR (Complete Response) | Disappearance of all target lesions | Complete disappearance of all measurable disease | Complete disappearance of all measurable and evaluable disease, no new lesions | Absent scitigraphic uptake | Normalized or undetectable | Normalized or undetectable | Normalizing calcitonin and CEA |
| PR (Partial Response) | ≥30% decrease in sum of longest diameters of target lesions | ≥50% decrease in the sum of products of perpendicular diameters (bidimensional) of measurable lesions | ≥50% decrease in sum of products of bidimensional lesions; no new lesions | Suppression of scitigraphic uptake | Significant sustained decrease (>50%) | Significant sustained decrease (>50%) | prolongation of calcitonin doubling time of at least 100% paired with decreasing calcitonin levels upon treatment |
| SD (Stable Disease) | Neither sufficient shrinkage for PR nor sufficient increase for PD | Between ≤50% decrease and ≤25% increase in lesion size | Neither sufficient shrinkage for PR nor sufficient increase for PD | Similar/comparable scinitgraphic uptake | Fluctuating or minor decrease/increase (<50%) | Fluctuating or minor decrease/increase (<50%) | Biochemical disease remains; imaging stable |
| PD (Progressive Disease) | ≥20% increase in sum of diameters or new lesions | ≥25% increase in sum of products of bidimensional lesions or new lesions | ≥50% increase in bidimensional lesion size or new lesions | Rising scintigraphic uptake | Progressive increase (>50–100%) | Progressive increase (>50–100%) | Biochemical and radiologic progression |

**Table S4:** Results of Methodological Quality evaluation by NIH evaluation tool

| Criteria | Question | Beukhof 2019 | Bodei 2003 | Bodei 2004 | Dadgar 2023 | Iten 2007 | Liu 2023 | Parghane 2020 | Satapathy 2020 | Vaisman 2015 | Valkema 2002 | Waldherr 2001 |
| --- | --- | --- | --- | --- | --- | --- | --- | --- | --- | --- | --- | --- |
| 1 | **Was the research question or objective in this paper clearly stated?** | **Yes** | **Yes** | **Yes** | **Yes** | **Yes** | **Yes** | **Yes** | **Yes** | **Yes** | **Yes** | **Yes** |
| 2 | **Was the study population clearly specified and defined?** | **Yes** | **Yes** | **Yes** | **Yes** | **Yes** | **Yes** | **Yes** | **Yes** | **Yes** | **Yes** | **Yes** |
| 3 | **Was the participation rate of eligible persons at least 50%?** | **Yes** | **Yes** | **Yes** | **Yes** | **Yes** | **Yes** | **Yes** | **Yes** | **Yes** | **Yes** | **Yes** |
| 4 | **Were all the subjects selected or recruited from the same or similar populations (including the same time period)? Were inclusion and exclusion criteria for being in the study prespecified and applied uniformly to all participants?** | **Yes** | **Yes** | **Yes** | **No** | **No** | **Yes** | **Yes** | **Yes** | **Yes** | **Yes** | **Yes** |
| 5 | **Was a sample size justification, power description, or variance and effect estimates provided?** | **No** | **No** | **No** | **No** | **No** | **No** | **No** | **X** | **No** | **No** | **No** |
| 6 | **For the analyses in this paper, were the exposure(s) of interest measured prior to the outcome(s) being measured?** | **Yes** | **Yes** | **Yes** | **Yes** | **Yes** | **Yes** | **Yes** | **Yes** | **Yes** | **Yes** | **Yes** |
| 7 | **Was the timeframe sufficient so that one could reasonably expect to see an association between exposure and outcome if it existed?** | **Yes** | **Yes** | **Yes** | **X** | **Yes** | **Yes** | **Yes** | **Yes** | **Yes** | **Yes** | **Yes** |
| 8 | **For exposures that can vary in amount or level, did the study examine different levels of the exposure as related to the outcome (e.g., categories of exposure, or exposure measured as continuous variable)?** | **No** | **Yes** | **No** | **Yes** | **No** | **Yes** | **Yes** | **No** | **X** | **No** | **No** |
| 9 | **Were the exposure measures (independent variables) clearly defined, valid, reliable, and implemented consistently across all study participants?** | **Yes** | **Yes** | **Yes** | **Yes** | **Yes** | **Yes** | **Yes** | **Yes** | **Yes** | **Yes** | **Yes** |
| 10 | **Was the exposure(s) assessed more than once over time?** | **No** | **Yes** | **No** | **No** | **No** | **Yes** | **Yes** | **Yes** | **No** | **No** | **No** |
| 11 | **Were the outcome measures (dependent variables) clearly defined, valid, reliable, and implemented consistently across all study participants?** | **Yes** | **Yes** | **Yes** | **Yes** | **Yes** | **Yes** | **Yes** | **Yes** | **Yes** | **Yes** | **Yes** |
| 12 | **Were the outcome assessors blinded to the exposure status of participants?** | **X** | **X** | **X** | **X** | **X** | **X** | **X** | **X** | **X** | **X** | **X** |
| 13 | **Was loss to follow-up after baseline 20% or less?** | **Yes** | **Yes** | **Yes** | **X** | **Yes** | **Yes** | **Yes** | **X** | **X** | **Yes** | **X** |
| 14 | **Were key potential confounding variables measured and adjusted statistically for their impact on the relationship between exposure(s) and outcome(s)?** | **No** | **No** | **No** | **No** | **No** | **No** | **Yes** | **Yes** | **No** | **No** | **No** |
| Overall | | **9** | **11** | **9** | **7** | **8** | **11** | **12** | **10** | **8** | **9** | **8** |
| Quality | | **Good** | **Good** | **Good** | **Fair** | **Fair** | **Good** | **Good** | **Good** | **Fair** | **Good** | **Fair** |

**Table S5:** PRRT in Late MTC: Present Constraints and Planned Improvements

| Current evidence situation | Impact on generalizability to “late MTC” | Planned/future directions |
| --- | --- | --- |
| Most available MTC PRRT data come from small, single-center, retrospective series, reflecting the rarity of MTC and difficulty in recruiting large, homogeneous cohorts. | Retrospective design increase information bias, so reported efficacy and safety may not fully represent the broader late-stage MTC population. | Conduct multicenter, prospectively designed studies to pool larger numbers of late MTC patients with standardized data collection, response assessment, and toxicity reporting. |
| Included patients often had heterogeneous SSTR expression, and earlier studies sometimes used planar/SPECT or qualitative criteria instead of uniform Krenning-based PET/SPECT thresholds. | Inconsistent SSTR selection criteria mix truly SSTR-avid tumors with borderline uptake, making it unclear how well outcomes extrapolate across the full spectrum of SSTR expression in late MTC. | Require that the entire measurable tumor burden shows high SSTR uptake (e.g., Krenning score ≥3 on modern SSTR PET/SPECT) to align with current SSTR PRRT eligibility concepts and improve internal validity. |
| Current data support feasibility and potential benefit of PRRT in late MTC with demonstrable SSTR expression but do not provide definitive, population-level estimates for the entire spectrum of late-stage disease. | Clinicians should view existing results as most applicable to late MTC patients with robust SSTR expression and appropriate prior treatments, not to all late MTC irrespective of SSTR profile. | Generate higher-level evidence (prospective, multicenter, with stringent SSTR criteria) to clarify the true magnitude of benefit and define the optimal integration of SSTR PRRT into late MTC treatment algorithms. |
